# Dual β-Lactam Combinations Highly Active against Mycobacterium abscessus Complex *In Vitro*

**DOI:** 10.1128/mBio.02895-18

**Published:** 2019-02-12

**Authors:** R. Pandey, L. Chen, C. Manca, S. Jenkins, L. Glaser, C. Vinnard, G. Stone, J. Lee, B. Mathema, E. L. Nuermberger, R. A. Bonomo, B. N. Kreiswirth

**Affiliations:** a Public Health Research Institute Tuberculosis Center, New Jersey Medical School, Rutgers University, Newark, New Jersey, USA; b Department of Pathology and Laboratory Medicine, Weill Cornell Medical Center, New York, New York, USA; c Department of Clinical Microbiology, Hospital of the University of Pennsylvania, Philadelphia, Pennsylvania, USA; d Pfizer, Groton, Connecticut, USA; e Department of Medicine, Johns Hopkins University, Baltimore, Maryland, USA; f Department of Epidemiology, Mailman School of Public Health, Columbia University, New York, New York, USA; g Medical Service and GRECC, Louis Stokes Cleveland Department of Veterans Affairs Medical Center, Cleveland, Ohio, USA; New York University School of Medicine; Texas Biomedical Research Institute; Baylor College of Medicine

**Keywords:** beta-lactamases, beta-lactams, multidrug resistance, *Mycobacterium abscessus*

## Abstract

As a consequence of a growing population of immunocompromised individuals, including transplant recipients and cystic fibrosis patients, there has been a dramatic increase in chronic infections caused by Mycobacterium abscessus complex (MABC) strains that are usually recalcitrant to effective antibiotic therapy. The recent rise of macrolide resistance in MABC has further complicated this clinical dilemma, dramatizing the need for novel agents. The repurposing of current antibiotics is one rapid path from discovery to patient care. In this study, we have discovered that dual β-lactams, and specifically the combination of ceftazidime with either ceftaroline or imipenem, are synergistic and have clinically relevant activities, with MIC_50_s of 0.25 (ceftaroline with 100 µg/ml ceftazidime) and 0.5 µg/ml (imipenem with 100 µg/ml ceftazidime) against clinical MABC isolates. Similar synergy was observed in time-kill studies against the M. abscessus ATCC 19977 strain using clinically achievable concentrations of either imipenem (4 µg/ml) or ceftaroline (2 µg/ml), as the addition of ceftazidime at concentrations of ≥50 µg/ml showed a persistent bactericidal effect over 5 days. Treatment of THP-1 human macrophages infected with three different M. abscessus clinical isolates supported the *in vitro* findings, as the combination of 100 µg/ml ceftazidime and 0.125 µg/ml ceftaroline or 100 µg/ml ceftazidime and 0.25 µg/ml imipenem dramatically reduced the CFU counts to near baseline levels of infection. This study’s finding that there is synergy between certain β-lactam combinations against M. abscessus infection provides optimism toward identifying an optimum dual β-lactam treatment regimen.

## Abstract (precis)

The emergence of chronic MABC infections among immunocompromised populations and their inherent and acquired resistance to effective antibiotic therapy have created clinical challenges in advancing patients for transplant surgery and treating those with disease. There is an urgent need for new treatment regimens, and the repurposing of existing antibiotics provides a rapid strategy to advance a laboratory finding to patient care. Our recent discoveries that dual β-lactams, specifically the combination of ceftazidime with ceftaroline or ceftazidime with imipenem, have significant *in vitro* MIC values and kill curve activities and are effective against infected THP-1 human macrophages provide optimism for a dual β-lactam treatment strategy against MABC infections. The unexpected synergistic activities reported in this study create a new path of discovery to repurpose the large family of β-lactam drugs.

## INTRODUCTION

The rise in the number of immunocompromised individuals has resulted in a large and clinically significant increase in the number of infections caused by Mycobacterium abscessus complex (MABC) strains (1–3). These rapidly growing nontuberculous mycobacteria (NTM) cause chronic infections in immunocompromised patients, including patients with cancer and transplant recipients, as well as in patients with chronic lung disease, such as cystic fibrosis (4, 5). In most cases the infections are resistant to most antituberculosis agents and to other major antibiotic classes (2). The recent recognition of macrolide resistance has further narrowed treatment options (2). Combination drug regimens, which may include a β-lactam, aminoglycoside, macrolide, linezolid, or tigecycline, are routinely prescribed, but fewer than one-half of patients with pulmonary disease achieve sputum culture conversion (6, 7). Regrettably, treatment failures and recurrences are frequently reported (7–9). With the rapidly growing number of vulnerable populations, there is an urgent need for novel treatment approaches that are more likely to achieve clinical remission.

Genomic analyses, although limited, have dramatically increased our knowledge regarding the phylogenetic relatedness among the MABC strains: M. abscessus subsp. *abscessus*, M. abscessus subsp. *massiliense*, and M. abscessus subsp. *bolletii* (10–12). Comparative genomics has revealed recombination among the subspecies and with other NTM species, thereby creating admixed strains that are associated with chronic colonization and lung infections (13). Mining of putative resistance genes identified the acquisition of *erm*41 and mutations in *rrl* (14–16) to be associated with the emergence of macrolide resistance; the *rrs* mutation was found to be associated with amikacin resistance, and the initial characterization of the M. abscessus complex β-lactamase (Bla_Mab_) and transpeptidases were found to be related to β-lactam resistance (17–19).

With the current focus on drug discovery for multidrug-resistant Gram-negative pathogens and the need to overcome the emergence of carbapenem resistance, there are now active pharmaceutical programs developing β-lactamase inhibitors and clinical research efforts investigating dual β-lactam therapy (20, 21). Currently, the MABC treatment guidelines include the use of the β-lactam cefoxitin (a cephalosporin) or imipenem (a carbapenem) (22–24). Although they are more stable than other β-lactams in the presence of the principal M. abscessus β-lactamase, Bla_Mab_, their clinical efficacy remains uncertain. Recent studies indicate that, unlike other marketed β-lactamase inhibitors, the new non-β-lactam agent avibactam effectively inhibits Bla_Mab_ and significantly improves the activity of other β-lactams against M. abscessus, including the new cephalosporin ceftaroline (17, 19, 25–27). Together with recent evidence that different β-lactams have differing potencies against specific M. abscessus transpeptidases, these results suggest that dual β-lactam regimens with or without avibactam may have greater activity than a single agent (7, 17, 28).

In this study, we collected 29 clinical M. abscessus complex respiratory isolates from different institutions. All isolates were characterized by whole-genome sequencing, and each was tested *in vitro* against ceftaroline and imipenem, alone and in combination with ceftazidime-avibactam, a newly marketed cephalosporin–β-lactamase inhibitor combination. Surprisingly, despite the poor activity of ceftazidime with or without avibactam, the dual β-lactam combinations of ceftazidime with either ceftaroline or imipenem showed promising synergistic activities against the MABC strains. This discovery may catalyze a new strategy of combining β-lactams to more effectively treat this highly debilitating chronic infection.

## RESULTS

### Bacterial strains and genomic characterization.

**FIG 1:**
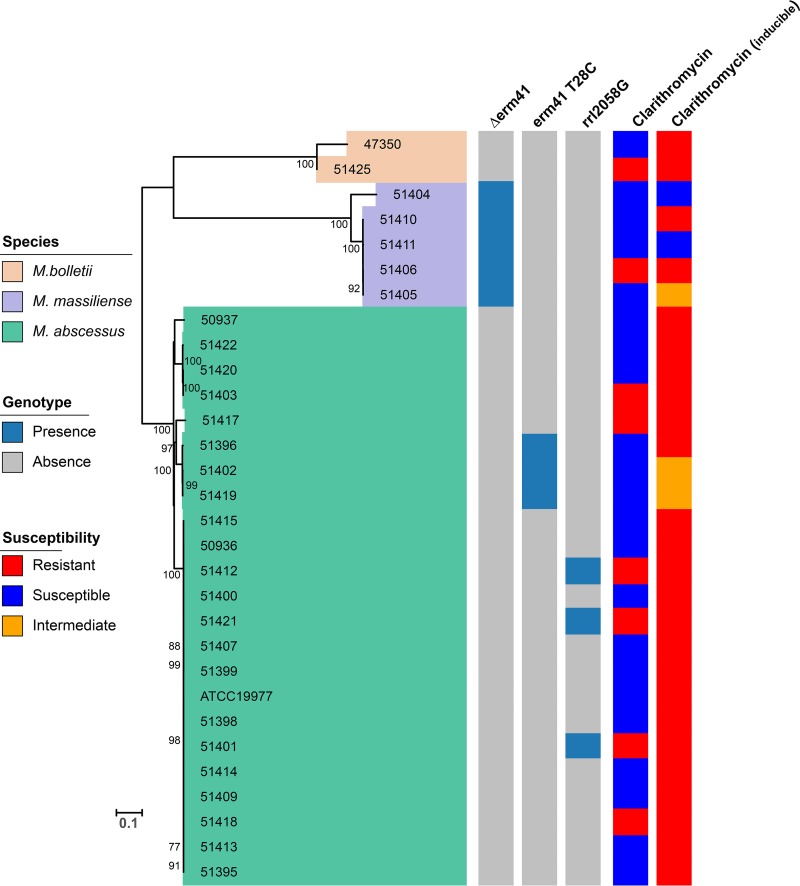
Phylogenetic analysis and susceptibility testing of *M. abscesses* complex isolates. The MICs of ≤2, 4, and ≥8 μg/ml were defined as susceptible, intermediate, and resistant to clarithromycin. Acquired clarithromycin resistance was determined at day 3, while inducible clarithromycin resistance was determined at day 14.

A total of 30 M. abscessus complex strains, including the reference strain ATCC 19977, were characterized by whole-genome sequencing and determined to the subspecies level as M. abscessus subsp. *abscessus*, M. abscessus subsp. *massiliense*, and M. abscessus subsp. *bolletii*. As shown in the phylogenetic tree in Fig. 1, all three subspecies were distinguished consistently with recent studies (12). Among them, 23 were typed as M. abscessus, 5 were *M. massiliense*, and 2 were *M. bolletii* strains.

The analysis of genes associated with macrolide resistance included the interrogation of *erm41* and *rrl*, in parallel with clarithromycin susceptibility testing. Acquired macrolide resistance in the MABC has been linked largely to the point mutations A2058 and A2059 in the *rrl* gene, encoding the peptidyltransferase domain of 23S rRNA (2, 29). An inducible macrolide resistance mechanism has also been described in MABC and involves the ribosomal methylase gene *erm41* (14). However, among the three MABC subspecies, *M. massiliense* carries a nonfunctional *erm41* gene and does not exhibit inducible macrolide resistance (14). In addition, an intact *erm41* gene with a T-to-C polymorphism at nucleotide (nt) 28 is associated with macrolide susceptibility. As shown in Fig. 1, a total of 20/23 M. abscessus strains contained the wild-type *erm41* gene. Among these 20 strains, 6 showed high-level macrolide resistance, which was explained by an *rrl* mutation in 3 strains, whereas a molecular explanation for the 3 others was not found. The remaining 14 strains with the wild-type *erm41* gene had inducible resistance. Three M. abscessus strains had the *erm41* gene with the T28C mutation, associated with macrolide susceptibility, but one displayed inducible clarithromycin resistance. All 5 *M. massiliense* strains had a truncated *erm41* gene, but only 3 were clarithromycin susceptible (*n* = 2) or intermediate (*n* = 1). The remaining 2 strains had inducible resistance or high-level resistance that was not explained by an *rrl* mutation. Finally, both *M. bolletii* strains had the wild-type *erm41* gene and showed inducible clarithromycin resistance, one of which showed high-level resistance without an *rrl* mutation. Analysis of the *rrl* gene identified macrolide resistance associated with the nt 2058 A→G mutation in 3 M. abscessus strains, and as predicted, all 3 strains expressed high-level resistance to clarithromycin (MIC > 128 µg/ml) (Fig. 1).

The above genomic analysis of a collection of recent MABC respiratory isolates from two medical centers identified the presence of all three subspecies of the MABC and the high prevalence of macrolide resistance with diverse and yet-uncharacterized molecular signatures. The identification of different resistance genotypes associated with either constitutive or inducible clarithromycin resistance dramatizes the plasticity of these subspecies and their adaptation from an environmental organism to a human pathogen in a susceptible host. These genomically characterized isolates then served as a panel of clinically relevant strains for susceptibility testing against different combinations of β-lactams and avibactam.

### Susceptibility of M. abscessus complex isolates to β-lactams and avibactam.

*In vitro* antibiotic susceptibility testing results for ceftaroline and imipenem, with or without avibactam or ceftazidime-avibactam, against 30 M. abscessus complex strains are presented in Table 1. The ceftaroline MICs ranged from 0.5 to >128 µg/ml, with an MIC_90_ and MIC_50_ of 32 and 16 µg/ml, respectively. In combination with 4 µg/ml avibactam, the MIC range improved to 0.06 to 4 µg/ml against M. abscessus while remaining at 16 to 32 µg/ml for *M. bolletii*; the MIC_90_ fell 8-fold to 4 µg/ml, and the MIC_50_ fell 16-fold to 1 µg/ml. Imipenem MICs ranged from 0.5 to 16 µg/ml, with MIC_90_ and MIC_50_ values of 8 and 2 µg/ml, respectively. In contrast to our findings with ceftaroline, we did not find a significant change when imipenem was combined with 4 µg/ml avibactam. However, combining either ceftaroline or imipenem with 100 µg/ml ceftazidime plus 4 µg/ml of avibactam, the only avibactam-containing formulation that is marketed for clinical use, reduced the MIC_90_ and MIC_50_ values to 1 and 0.25 µg/ml, respectively, for ceftaroline, and 2 and 0.5 µg/ml, respectively, for imipenem, representing a 4-fold MIC reduction compared to those of combinations with avibactam alone (Table 1).

**TABLE 1:** Antimicrobial activities of ceftaroline or imipenem alone and in combination with ceftazidime and/or avibactam against MABC isolates^*a*^

Strain	Species	MIC (µg/ml)									
		CAZ	CAZ + AVI4	CFT	CFT + AVI4	CFT + CAZ100	CFT + CAZ100 + AVI4	IMI	IMI + AVI4	IMI + CAZ100	IMI + CAZ100 + AVI4
51425	M. bolletii	>1024	>1024	32	32	16	32	1	1	0.5	0.5
47350	M. bolletii	32	32	16	16	0.5	0.5	2	2	1	0.5
51404	M. massiliense	128	128	8	0.25	0.25	0.25	16	16	0.5	0.5
51405	M. massiliense	256	256	16	1	0.5	0.5	8	8	1	2
51406	M. massiliense	128	128	8	1	1	1	16	8	1	2
51410	M. massiliense	256	256	4	0.5	1	1	0.5	0.25	1	1
51411	M. massiliense	256	256	4	1	0.125	0.125	2	2	0.5	0.5
50936	M. abscessus	256	128	8	1	0.25	0.25	4	0.5	0.5	0.5
50937	M. abscessus	512	512	4	0.5	0.25	0.25	2	2	2	2
51395	M. abscessus	512	512	32	0.5	0.125	0.125	2	2	0.06	0.06
51396	M. abscessus	256	256	2	1	1	1	8	8	0.06	0.06
51398	M. abscessus	128	128	8	0.5	0.125	0.125	4	2	0.5	0.5
51399	M. abscessus	128	128	8	0.5	0.5	0.5	16	8	0.125	0.125
51400	M. abscessus	128	128	128	1	0.125	0.125	4	2	0.5	0.5
51401	M. abscessus	256	256	16	2	0.125	0.125	2	2	0.125	0.125
51402	M. abscessus	256	128	16	2	1	1	8	8	0.125	0.125
51403	M. abscessus	128	128	8	0.5	0.25	0.25	8	4	0.5	0.5
51407	M. abscessus	256	256	4	1	0.25	0.25	2	2	0.25	0.25
ATCC 19977	M. abscessus	512	512	32	0.5	0.125	0.125	8	4	0.25	0.25
51409	M. abscessus	128	128	16	0.5	0.25	0.25	0.5	0.5	2	1
51412	M. abscessus	256	256	8	0.5	0.5	1	0.5	0.25	0.125	0.5
51413	M. abscessus	256	256	>128	4	2	1	0.5	0.125	0.5	0.5
51414	M. abscessus	256	128	32	1	1	2	0.5	0.25	0.5	0.5
51415	M. abscessus	256	256	4	1	0.25	0.25	0.5	0.5	1	1
51417	M. abscessus	512	128	32	4	2	2	0.5	0.25	1	1
51418	M. abscessus	256	256	32	2	2	1	0.5	0.5	1	1
51419	M. abscessus	8	8	16	0.5	0.25	0.25	1	0.5	1	1
51420	M. abscessus	256	256	0.5	0.06	0.25	0.25	1	0.5	2	2
51421	M. abscessus	256	256	32	2	0.5	0.5	1	0.5	0.25	0.25
51422	M. abscessus	256	256	16	1	1	1	1	1	1	1

MIC_50_		256	256	16	1	0.25	0.25	2	2	0.5	0.5
MIC_90_		512	512	32	4	2	1	8	8	1	2

a CAZ, ceftazidime; AVI, avibactam; CFT, ceftaroline; IMI, imipenem; CAZ100, 100 µg/ml ceftazidime; AVI4, 4 µg/ml avibactam.

Although the *in vitro* activity of ceftazidime alone is limited against MABC strains, with an MIC_50_ of 256 µg/ml, the results in Table 1 provide compelling evidence that this cephalosporin is very effective in combination with both imipenem and ceftaroline, with or without avibactam. Indeed, the MICs of ceftaroline or imipenem in the presence of ceftazidime were the same as, or within 1 dilution of, the MICs in the presence of both ceftazidime and avibactam, indicating that the synergistic or additive effect of adding ceftazidime and avibactam to imipenem is driven almost entirely by ceftazidime rather than avibactam. To confirm this finding and elucidate the dose-response effect of ceftazidime in the absence of avibactam, we determined the MICs of imipenem and ceftaroline against 3 M. abscessus strains in the presence of various subinhibitory concentrations of ceftazidime (10, 50, and 100 µg/ml). As shown in Table 2, the absence of avibactam did not alter the synergistic activity between the dual β-lactam combinations, and decreasing the concentration of ceftazidime to as low as 10 µg/ml still improved the MICs of both imipenem and ceftaroline, although there was some concentration-dependent effect of ceftazidime.

**TABLE 2:** Activities of ceftaroline or imipenem alone and in combination with increasing concentrations of ceftazidime and/or avibactam against M. abscessus complex isolates^*a*^

Strain	Species	MIC (μg/ml)										
		CAZ + AVI4	CFT	CFT + CAZ10	CFT + CAZ50	CFT + CAZ100	CFT + CAZ100 + AVI4	IMI	IMI + CAZ10	IMI + CAZ50	IMI + CAZ100	IMI + CAZ100 + AVI4
ATCC 19977	M. abscessus	512	32	0.25	0.125	0.125	<0.25	8	2	0.25	0.125	0.25
51412	M. abscessus	256	8	0.5	0.5	0.5	1	0.5	0.25	0.06	0.06	0.5
51403	M. abscessus	128	8	1	0.25	0.125	0.25	8	1	0.5	0.5	0.5

a CAZ, ceftazidime; AVI, avibactam; CFT, ceftaroline; IMI, imipenem; CAZ10, 10 µg/ml ceftazidime; CAZ50, 50 µg/ml ceftazidime; CAZ100, 100 µg/ml ceftazidime; AVI4, 4 µg/ml avibactam.

### *In vitro* time-kill studies with imipenem and ceftaroline alone and in combination with ceftazidime.

**FIG 2:**
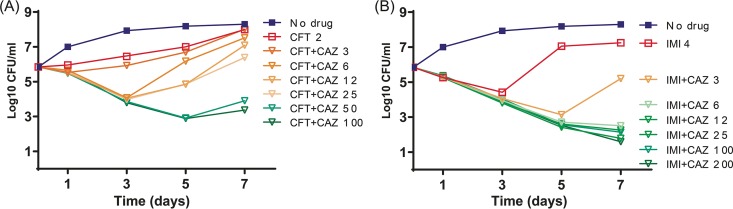
*In vitro* time-kill studies with ceftaroline (A) or imipenem (B), either alone or in combination with different concentrations of ceftazidime. CAZ, ceftazidime; CFT, ceftaroline; IMI, imipenem.

The ATCC 19977 strain was used for *in vitro* time-kill assays evaluating the activities of ceftaroline and imipenem, with and without increasing concentrations of ceftazidime. Consistently with the concentration-dependent synergy observed with *in vitro* MIC testing, the addition of increasing (but subinhibitory) concentrations of ceftazidime improved the bactericidal activities of both drugs. As shown in Fig. 2A, ceftaroline alone at 2 µg/ml had weak inhibitory activity, and the addition of ceftazidime at concentrations as low as 6 µg/ml significantly resulted in increased killing over the first 3 days (*P* < 0.05), while ceftazidime 50 µg/ml enabled bactericidal activity for 5 days (*P* < 0.01). Imipenem alone at 4 µg/ml resulted in a bactericidal effect over the first 3 days of exposure, followed by an increasing CFU count (*P* < 0.01). Addition of ceftazidime concentrations of ≥6.25 µg/ml improved the activity of imipenem and maintained its bactericidal effect over 7 days (*P* < 0.01) (Fig. 2B).

### Intracellular activities of ceftaroline and imipenem alone or in combination with avibactam or avibactam-ceftazidime.

**FIG 3:**
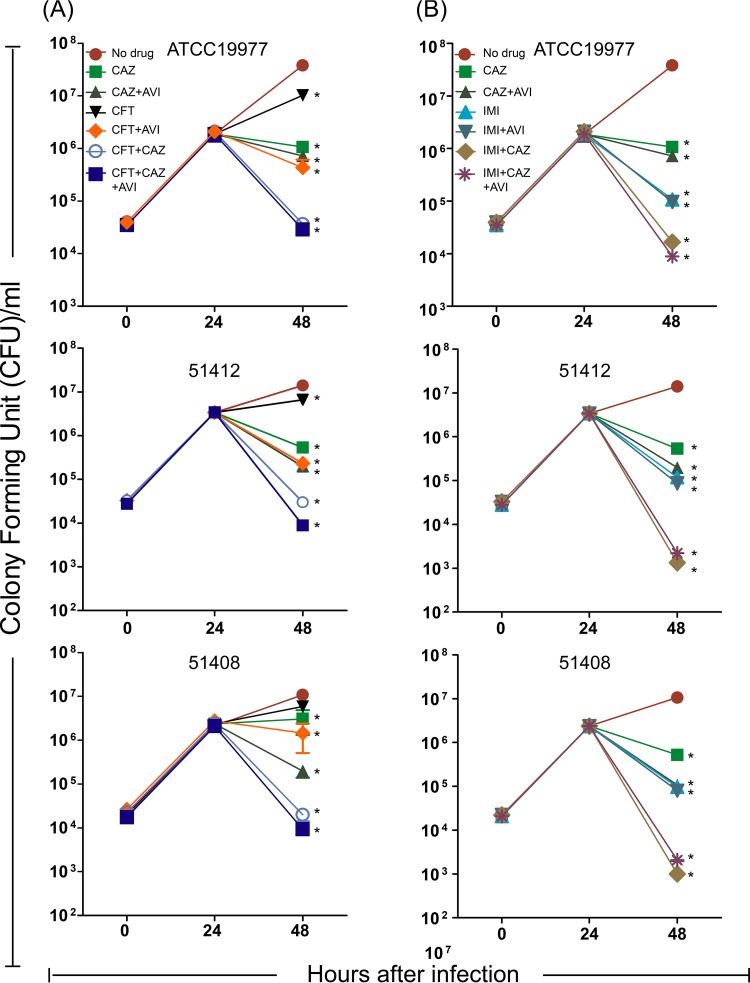
Intracellular activity of ceftaroline (A) or imipenem (B), either alone or in combination with avibactam or avibactam-ceftazidime. Ceftaroline (CFT; 0.125 μg/ml), imipenem (IMI; 0.25 μg/ml), ceftazidime (CAZ; 100 μg/ml), or avibactam (AVI; 4 μg/ml) alone or in different combinations were used. *, compared with no drug group, *P* < 0.05.

Based on the results in axenic culture, we evaluated the intracellular activities of the dual β-lactam combinations with and without avibactam against 3 selected M. abscessus strains in a THP-1 macrophage assay. Figure 3A shows the CFU results treating with ceftaroline alone and in combination, and Fig. 3B shows the parallel results treating with imipenem. Consistently with the results in axenic cultures, imipenem (0.25 µg/ml) showed better intracellular activity than ceftaroline (0.125 µg/ml) and the addition of avibactam (4 µg/ml) only improved the activity of ceftaroline. In contrast to the results in axenic cultures, ceftazidime (100 µg/ml) alone had static or weakly bactericidal intracellular activity, and this slightly improved with the addition of avibactam. The most important finding, and one that supports the results in axenic cultures, was that ceftazidime-ceftaroline and ceftazidime-imipenem had excellent activity in the absence of avibactam, reducing CFU counts by 2 to 3 log_10_ over a 24-h treatment period (*P* < 0.01).

### Determination of the gene expression of *bla*_Mab_.

**FIG 4:**
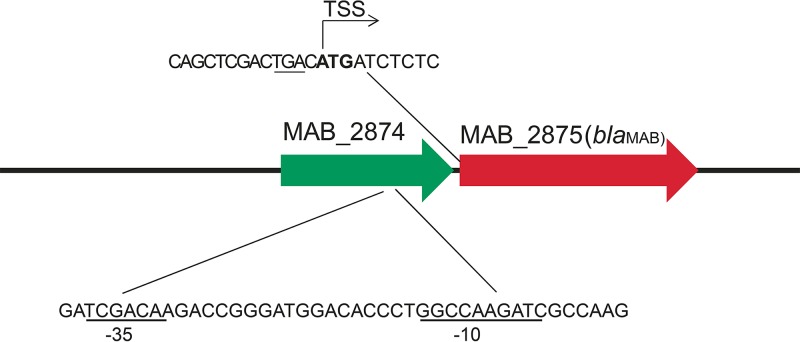
Genetic environment of the *bla*_Mab_ gene. The promoter sequence of *bla*_Mab_ is embedded in the upstream gene (locus tag MAB_2874). The putative −35 and −10 promoter sequences are underlined in the lower sequence. TSS, transcription start site. The stop codon (TGA) of MAB_2874 is underlined in the upper sequence, while the start codon (ATG) of *bla*_Mab_ is shown in bold.

MABC isolates harbor a chromosomally encoded β-lactamase, Bla_Mab_, which is primarily responsible for the poor efficacies of β-lactams against MABC. Analysis of the *bla*_Mab_ gene showed that the 30 MABC isolates had the same upstream isomerase gene (locus number MAB_2874), which was separated by a single nucleotide (a single nucleotide between the stop codon of the isomerase gene and the start codon of *bla*_Mab_) (Fig. 4). The mapping of the transcriptional start site by rapid amplification of 5′ cDNA ends (5′RACE) revealed that the *bla*_Mab_ ATG translational start site is also the transcriptional start site. The −35 and −10 promoters of *bla*_Mab_ are embedded in the 3′ end of upstream gene MAB_2874, which is conserved among all MABC isolates (Fig. 4). In general, the *bla*_Mab_ sequences correlated with their subspecies assignment. However, *bla*_Mab_ in M. abscessus strain 51403 displayed nearly identical sequences (1 single nucleotide polymorphism [SNP] difference) to those of *M. bolletii* strains 47350 and 51425, likely due to the recombination of the *bla*_Mab_ region among different subspecies of the M. abscessus complex (see Fig. S1 in the supplemental material).

10.1128/mBio.02895-18.1 FIG S1 Alignment of Bla_Mab_ amino acid sequences from 30 M. abscessus complex isolates. Download FIG S1, PDF file, 0.1 MB. Copyright © 2019 Pandey et al. 2019 Pandey et al. This content is distributed under the terms of the Creative Commons Attribution 4.0 International license.

**FIG 5:**
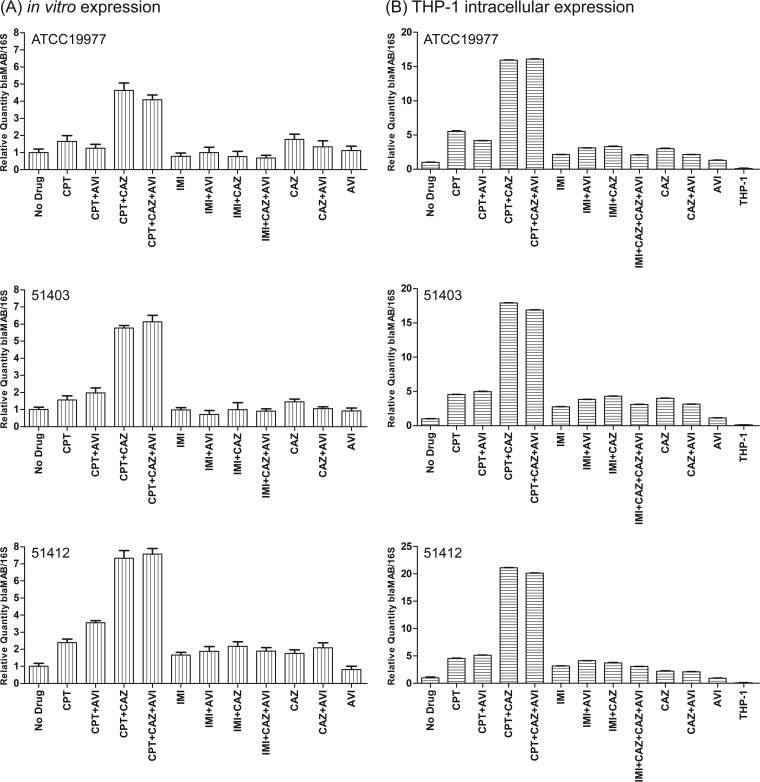
*In vitro* (A) and intracellular (B) *bla*_Mab_ expression during exposure to different combinations of ceftaroline or imipenem, either alone or in combination with ceftazidime and/or avibactam. Ceftaroline (0.125 μg/ml), imipenem (0.25 μg/ml), ceftazidime (100 μg/ml), or avibactam (4 μg/ml) alone or in different combinations was used.

Expression of both the *bla*_Mab_ gene and upstream gene MAB_2874 was analyzed at the transcriptional level in 3 M. abscessus strains, including M. abscessus ATCC 19977. The strains were grown *in vitro*, with or without drug, and both genes were evaluated. Compared to levels in the absence of drugs, the relative quantity of the *bla*_Mab_ transcript was elevated 4- to 7-fold in all 3 M. abscessus strains when the cells were grown in the presence of 0.25 µg/ml ceftaroline plus 100 µg/ml ceftazidime, with or without 4 µg/ml avibactam (*P* < 0.01) (Fig. 5A). In contrast, there was no significant change in the relative quantities of the *bla*_Mab_ transcript in the presence of imipenem, alone or in combination with avibactam or ceftazidime-avibactam (*P* > 0.05). The expression of the upstream gene MAB_2874 was not affected by the presence of any drug tested alone or in combination (data not shown).

Consistently with the *in vitro* findings, RNA isolated from the same 3 M. abscessus strains when grown in THP-1 cells in the presence of the same antibiotics showed that the relative quantities of the *bla*_MAb_ transcript were elevated >15-fold in all 3 strains when grown in the presence of 0.25 µg/ml ceftaroline plus 100 µg/ml ceftazidime, with or without 4 µg/ml avibactam (Fig. 5B) (*P* < 0.01). These results suggest a complex *bla*_Mab_ induction profile under multiple β-lactam–β-lactamase inhibitor challenges and that Bla_Mab_ itself is insufficient to explain the dual-β-lactam effects, indicating that additional mechanisms may be involved. Understanding *bla*_Mab_ gene control, which appears to be upregulated in response to cell stress, will be important in considering the use of β-lactams and β-lactam inhibitors to challenge infections with this complex species.

## DISCUSSION

Currently, combination therapies are recommended to treat M. abscessus pulmonary infections with drug classes that include a macrolide (e.g., clarithromycin or azithromycin), an aminoglycoside (e.g., amikacin), and a β-lactam (e.g., cefoxitin or imipenem) (7, 22–24). However, the recognition that macrolide resistance, now present in over 50% of clinical isolates (30), dramatically alters treatment outcomes has fueled the search for better treatment options, since the clearance of MABC from sputum ranges from 45 to 75% in patients with macrolide-susceptible strains but ranges from only 5 to 15% in patients with resistant strains (5).

Cefoxitin and imipenem, which are relatively stable in the presence of Bla_Mab_, are the β-lactams currently recommended in treatment guidelines for M. abscessus infections; however, our knowledge regarding the efficacy of including either agent remains limited. In addition, other β-lactams are more efficiently hydrolyzed by Bla_Mab_, and established β-lactamase inhibitors, including clavulanate, sulbactam, and tazobactam, do not significantly improve their *in vitro* activities, as there is limited inhibition against the M. abscessus β*-*lactamase.

New β-lactams and β-lactam inhibitors developed for other resistant bacterial infections are worthy of consideration for repurposing against M. abscessus infection. For example, Dubee et al. (25) showed that the recently approved non-β-lactam β-lactamase inhibitor avibactam is a potent inhibitor of the *M. abscessus* β-lactamase and that, at high concentrations, it enhanced the activity of amoxicillin against M. abscessus-infected macrophages and zebrafish. However, avibactam at 4 µg/ml, the concentration recommended for drug susceptibility testing in combination with ceftazidime against other pathogens (31), did not improve the *in vitro* activity of either cefoxitin or imipenem. Likewise, Lefebvre and coworkers (19) performed time-kill studies with imipenem and cefoxitin against M. abscessus and a *bl*a_Mab_-deficient mutant and concluded that the M. abscessus β-lactamase does not affect the killing activity of either β-lactam. However, they did observe that avibactam at 4 µg/ml enhanced the bactericidal activity of ceftaroline, a broad-spectrum new-generation cephalosporin developed for methicillin-resistant Staphylococcus aureus infections. Prior *in vitro* testing against 10 M. abscessus complex isolates showed a ceftaroline MIC range from 2 to 64 µg/ml, with an MIC_50_ of 4 µg/ml (19), and improved ceftaroline activity with the addition of 4 µg/ml avibactam, which decreased the MIC range to 0.5 to 2 µg/ml, with an MIC_50_ of 1 µg/ml (32, 33). Kinetic studies with the purified Bla_Mab_ enzyme revealed that ceftaroline is efficiently hydrolyzed (*k*_cat_/*K_m_* = 5.5 × 10^4^ M^−1^ s^−1^), and therefore, the MIC decrease in the presence of avibactam provides evidence that the inhibitor alters the enzymatic activity of the M. abscessus β-lactamase (32). However, the M. abscessus β-lactamase also efficiently hydrolyzes imipenem (*k*_cat_/*K_m_* = 3 × 10^4^ M^−1^ s^−1^), but in this case, the addition of avibactam does not improve the *in vitro* activity.

Our *in vitro* results with 30 M. abscessus complex strains confirm and extend prior observations, as imipenem had lower MICs than ceftaroline and the addition of avibactam had no significant impact on imipenem MICs but significantly improved ceftaroline MICs (Table 1). Because avibactam is clinically available only in combination with ceftazidime, we evaluated the *in vitro* activities of ceftaroline and imipenem against these 30 strains in the presence of both 100 µg/ml ceftazidime and 4 µg/ml avibactam. Surprisingly, and one of the novel findings in this study, the addition of ceftazidime-avibactam together further decreased the MIC_50_s of ceftaroline and imipenem by 4-fold (compared to their MIC_50_s after the addition of avibactam alone) to 0.25 and 0.5 µg/ml, respectively. Furthermore, we found this effect to be driven primarily by ceftazidime rather than avibactam, despite the very poor *in vitro* activity of ceftazidime alone against M. abscessus complex strains. In the presence of 100 µg/ml ceftazidime, the MICs of ceftaroline and imipenem ranged mainly from 0.06 to 0.5 µg/ml. Even when combined with a lower concentration of 10 µg/ml ceftazidime, ceftaroline maintained MICs of ≤0.5 µg/ml, the FDA-approved breakpoint for ceftaroline against S. aureus, and imipenem maintained MICs of ≤2 µg/ml, below its recommended breakpoint against M. abscessus, albeit in a different medium. These findings indicate significant potential for target attainment at commonly recommended doses of these β-lactams.

Similar levels of synergy were observed in the time-kill studies against the M. abscessus ATCC 19977 strain using clinically achievable concentrations of either imipenem (4 µg/ml) or ceftaroline (2 µg/ml). The addition of ceftazidime at 6 µg/ml with imipenem resulted in a 3-log_10_ reduction in the number of CFU per milliliter over 7 days. The same ceftazidime concentration increased ceftaroline activity over 3 days, but higher concentrations of ≥50 µg/ml were required for a more persistent bactericidal effect (Fig. 2A). Target attainment analyses (34) indicate a high probability of exceeding ceftazidime concentrations of 6 µg/ml for at least 50% of the dosing interval and of exceeding maximum concentration (*C*_max_) values of 50 µg/ml, associated with the dose of 2 g intravenously every 8 h, currently recommended to treat patients with Gram-negative infections. Even greater exposures may be possible with extended infusion times.

The results observed with dual β-lactam combinations in axenic cultures and time-kill studies were further supported by the THP-1 macrophage infection studies with three different clinical isolates of M. abscessus. The addition of avibactam did not significantly improve ceftazidime activity in all experiments; however, consistently with the *in vitro* findings, the combination of 100 µg/ml of ceftazidime and 0.125 µg/ml ceftaroline or of 100 µg/ml ceftazidime and 0.25 µg/ml imipenem dramatically reduced the CFU counts to near baseline levels of infection. Unlike in other reports (19) which used 16 µg/ml avibactam in macrophages and 50 µg/ml in zebrafish embryos, in our experiments, the addition of avibactam at 4 µg/ml provided limited improvement except when added to ceftaroline alone.

We observed that the expression of the M. abscessus β-lactamase gene is elevated in infected macrophages, unlike in broth, consistent with the current literature (19). Testing the same strains against β-lactams, both in THP-1 cells and in broth cultures, revealed that *bla*_Mab_ transcription was consistently and significantly elevated when exposed to ceftaroline and ceftazidime in combination. The increased expression of Bla_Mab_ in macrophages and in the presence of ceftaroline and ceftazidime suggests that the β-lactamase plays a role in peptidoglycan remodeling under these stressful conditions and, in so doing, may also reduce β-lactam activity. The unique combined effect of ceftaroline and ceftazidime on Bla_Mab_ suggests that it might be a function of the combined inhibition of a specific pattern of enzymes related to peptidoglycan synthesis and remodeling.

In this study, we used whole-genome sequencing to determine to the subspecies level 30 clinical isolates from the M. abscessus complex and to genotype macrolide resistance. Phylogenetic analysis showed that all three subspecies are represented and that the strains are genetically diverse. The majority showed phenotypic resistance to clarithromycin, which correlated with the acquisition of the *erm41* gene and/or mutations in the *rrl* gene; however, we did observe inconsistencies between genotypes and phenotypes that suggest that other genetic mechanisms might contribute to macrolide resistance. The emergence and increase in macrolide resistance heightens the need for new therapies and furthers the potential promise of a dual β-lactam approach.

There is increasing interest in combining different β-lactams, with and without β-lactam inhibitors, for their additive or synergistic effects against different pathogens. The combination of penicillins and ceftriaxone has been shown to be efficacious against Enterococcus faecalis endocarditis, and the combination of aztreonam with ceftazidime-avibactam has been successfully used to treat infection with metallo-β-lactamase-producing Enterobacteriaceae (35–38). Whether combining ceftazidime with ceftaroline or imipenem based on our promising *in vitro* susceptibility and time-kill data will prove to be an effective addition to current treatment regimens will require further animal and/or *in vitro* efficacy studies to further our knowledge. However, the overall finding that certain dual β-lactam combinations are synergistic provides optimism toward finding alternative treatment options to overcome the emergence of macrolide resistance among MABC strains.

In summary, our results provide positive evidence to pursue the use of dual β-lactam combinations in the treatment of MABC infections and, perhaps, their potential role in treating other NTM infections. Given the various combinations of possible β-lactam drugs available and the growing pipeline of novel β-lactamase inhibitors, there is a unique opportunity to develop a robust program to identify novel treatment regimens for MABC infections.

## MATERIALS AND METHODS

### Bacterial strains and growth conditions.

M. abscessus reference strain ATCC 19977 and 29 MABC clinical isolates from patients at the Weill Cornell Medical School and the Hospital of the University of Pennsylvania were grown in Middlebrook 7H9 broth (BD-Difco, Le Pont de Claix, France) supplemented with 0.2% glycerol, 10% (vol/vol) oleic acid-albumin-dextrose-catalase (OADC; BD-Difco) and 0.05% (vol/vol) Tween 80 (Sigma-Aldrich; 7H9sB). Cultures were incubated at 37°C with constant shaking (150 rpm) overnight to reach an optical density at 600 nm (OD_600_) of 0.5 to 0.7.

### Antibiotics.

Clarithromycin (Sigma), ceftaroline (IHMA Inc.), imipenem (Sigma), ceftazidime (Sigma), and avibactam (IHMA Inc.) were used alone or in combination. Stock solutions of amikacin, imipenem, and avibactam (10 mg/ml, final concentration) were prepared in sterile water. Stock solutions of clarithromycin were prepared in acetone, and stock solutions of ceftazidime and ceftaroline were prepared in dimethyl sulfoxide (DMSO), filter sterilized, and diluted in 7H9sB to obtain the desired working concentrations.

### Antibiotic susceptibility testing.

MICs were determined by the broth microdilution method in 96-well microtiter plates and modified from the CLSI guidelines (39). Ceftaroline and imipenem were also tested in combination with avibactam (4 µg/ml) and/or ceftazidime (100 µg/ml). Briefly, approximately 5 × 10^5^ CFU/ml was inoculated into 7H9sB containing 2-fold dilutions of the antibiotics of interest (concentration range from 0.125 µg/ml to 128 µg/ml). Microtiter plates, except those with clarithromycin, were incubated at 37°C for 48 h. For clarithromycin, microtiter plates were incubated at 30°C for 14 days and the MIC was determined on days 3, 7, 9, and 14. The MIC was defined as the lowest antibiotic concentration that prevented visible bacterial growth, and for susceptibility to amikacin and clarithromycin, we used the CLSI breakpoint recommendations (39). Acquired clarithromycin resistance was determined at day 3, while inducible clarithromycin resistance was determined at day 14.

### Evaluation of drug efficacy in THP-1 cells.

Infection of human THP-1 cells (ATCC) was performed to evaluate the intracellular activities of antibiotics against MABC isolates. Briefly, THP-1 cells were cultured at the concentration of 1 × 10^5^ per well in 96-well tissue culture plates and differentiated with 50 nM phorbol myristate acetate (PMA) for 24 h prior to infection. Cells were infected at a low multiplicity of infection (MOI) of 1:10 (bacterium/cell ratio). After 4 h of incubation at 37°C in a 5% CO_2_ atmosphere, cells were washed three times with warm phosphate-buffered saline (PBS) to remove extracellular bacteria and incubated for an additional 20 h in fresh RPMI 1640 supplemented with 10% human serum (R10). At 24 h postinfection, cells were washed three times with warm PBS prior to antibiotic treatment with ceftaroline (0.125 µg/ml), imipenem (0.25 µg/ml), ceftazidime (100 µg/ml), or avibactam (4 µg/ml) alone or in different combinations. Infected control cells received culture medium alone. At the time points postinfection indicated in the figures, cells were lysed with 0.05% sodium dodecyl sulfate (SDS) and numbers of CFU were determined by plating serial dilutions of the lysates on 7H10 plates. Cell viability was evaluated by a trypan blue exclusion assay prior to and following infection and/or treatment at each time point analyzed. Infection of THP-1 cells resulted in cell viability of 95% at the 48-h time point, while exposure of the cells to the various drugs did not affect cell viability (100%).

### Macrophage infection for bacterial RNA preparation.

THP-1 cells were cultured, infected, and treated as described above. After 24 h in the presence of the drug, cells from 3 wells were processed for bacterial RNA extraction.

### Transcriptional start site mapping.

The transcriptional start site of MAB_2875 (*bla*_Mab_) was identified by 5′ rapid amplification of cDNA ends (5′RACE) using a 5′/3′ RACE kit (Roche) and with the gene-specific primers *bla*_Mab_ R1 (CAAGCGCCGAAGGCCCGCAG) and *bla*_Mab_ R2 (AATCGTCGGCTTACCTTTGG) according to the manufacturer’s instructions.

### Time-kill studies.

M. abscessus ATCC 19977 was grown in 7H9sB overnight and inoculated at a concentration of approximately 5 × 10^5^ CFU/ml into wells containing 7H9sB and either imipenem (4 µg/ml) or ceftaroline (2 µg/ml), alone or in combination with increasing concentrations of ceftazidime from 3.125 to 200 µg/ml. At time zero and after 1, 3, 5, and 7 days of incubation, aliquots were removed, serially diluted, and plated on 7H11 medium for CFU quantification. Colonies were counted after 7 days of incubation at 37°C.

### RNA isolation from *in vitro* cultures and quantitative real-time PCR (qRT-PCR).

M. abscessus strains were grown to early logarithmic phase in Middlebrook 7H9 supplemented with 0.2% glycerol, 0.05% Tween 80, and ADS (0.5% bovine serum albumin [BSA], 0.2% dextrose, and 0.085% NaCl) without drug or in the presence of ceftaroline alone (0.125 µg/ml), imipenem alone (0.25 µg/ml), ceftaroline (0.25 µg/ml) with ceftazidime-avibactam (100/4 µg/ml), or imipenem (0.25 µg/ml) with ceftazidime-avibactam (100/4 µg/ml). Cells were collected by centrifugation, and pellets were resuspended in 1 ml TRI reagent, immediately transferred to a tube containing 0.5 ml 200-µm zirconia beads (Sigma-Aldrich), and disrupted by two 1-min pulses in a BeadBeater. RNA was purified using RNeasy columns by following the manufacturer instructions (Qiagen). The quality and quantity of purified total RNA were estimated using a NanoDrop spectrophotometer (NanoDrop, Wilmington, DE).

PCR was performed in a Bio-Rad i-cycler using Brilliant II SYBR Green qRT-PCR 1-step master mix (Agilent Technologies). PCR conditions were identical for all reactions. The 15-µl reaction mixture contained 50 ng of the total RNA templet, 7.5 µl of SYBR Green master mix, 0.3 µl primer mix (10 pmol of each primer), and nuclease-free water. The reverse transcriptase was incubated at 50°C for 30 min. After 10 min at 95°C to activate the DNA polymerase, a set of 40 cycles of 30 s at 95°C, annealing at 55°C for 30 s, and elongation at 72°C for 30 s was run. Prior to determining the dissociation curve, the reaction mixtures were incubated for 1 min at 95°C to denature the PCR and then ramped down to 55°C. For the dissociation curve, the temperature ramped from 55°C to 95°C. Primers used for qRT-PCR are listed in Table S1 in the supplemental material. Transcript abundance was normalized to the amount of 16S rRNA.

10.1128/mBio.02895-18.2 TABLE S1 Primers used in this study. Download Table S1, DOCX file, 0.01 MB. Copyright © 2019 Pandey et al. 2019 Pandey et al. This content is distributed under the terms of the Creative Commons Attribution 4.0 International license.

### M. abscessus RNA from macrophage cultures.

Macrophages infected with M. abscessus were lysed with 0.4 ml guanidinium thiocyanate-based buffer (25 mM sodium citrate–4 M guanidine thiocyanate–0.5% *N*-lauryl sarcosine–0.125 M mercaptoethanol–0.5% Tween 80, pH 7.0) and vortexed for 5 min to shear the eukaryotic DNA. Samples were centrifuged for 15 min at 4,000 × *g*, and bacterial pellets were washed with 0.1 ml of guanidinium thiocyanate-based buffer and centrifuged for 15 min at 4,000 × *g*. Finally, bacterial pellets were suspended in 1 ml of TRI reagent and processed as described for the RNA isolation from *in vitro* cultures.

### Whole-genome sequencing.

Isolates were grown in Middlebrook medium, and genomic DNA was extracted using a Qiagen QIAamp DNA minikit. The genomes were sequenced using an Illumina NextSeq platform by 150-bp paired-end reads. The Illumina reads were *de novo* assembled using SPAdes (40), followed by resistance gene (e.g., *bla*_Mab_, *erm41*, and *rrl*) examination using BLAST. For the phylogenetic analysis, the Illumina raw reads were mapped to a reference genome (ATCC 19977) using snippy (https://github.com/tseemann/snippy). Prophages were predicted using PHASTER (41), and repeated regions were examined using MUMmer (42), while regions of recombination were detected using Gubbins (43). SNPs among prophages and repeated or recombination regions were filtered using vcftools (http://vcftools.sourceforge.net/). Concatenated core genome SNP sites were extracted from the recombination-free alignment and a maximum-likelihood phylogenetic tree was inferred from the resulting SNP alignment in RAxML v8.2.4 (44).

### Statistical analyses.

The significance of differences between study groups was determined with Student's *t* test. All *P* values were based on two-tailed tests of significance. A *P* value of ≤0.05 was considered significant. All computations were performed with GraphPad Prism 7.

### Data availability.

The raw reads of this study have been uploaded to NCBI BioProject number PRJNA353361.
