# Supplementary material for: Dual β-Lactam Combinations Highly Active against Mycobacterium abscessus Complex In Vitro
Source: mBio. 2019 Feb 12;10(1):e02895-18. doi: 10.1128/mBio.02895-18 (PMC6372805; doi:10.1128/mBio.02895-18)
Supplement: FIG S1 [file mBio.02895-18-sf001.pdf]

```
                   10        20        30        40        50        60
          ....|....|....|....|....|....|....|....|....|....|....|....|
ATCC19977 MISRRALLVGGVSAVGVVAAGCSRNGNRRPAPDELASLEKDFGGRIGVYALDTGSGDTVG
51395     MISRRALLVGGVSAVGVVAAGCSRNGNRRPAPDELASLEKDFGGRIGVYALDTGSGDTVG
51412     MISRRALLVGGVSAVGVVAAGCSRNGNRRPAPDELASLEKDFGGRIGVYALDTGSGDTVG
51400     MISRRALLVGGVSAVGVVAAGCSRNGNRRPAPDELASLEKDFGGRIGVYALDTGSGDTVG
51407     MISRRALLVGGVSAVGVVAAGCSRNGNRRPAPDELASLEKDFGGRIGVYALDTGSGDTVG
50936     MISRRALLVGGVSAVGVVAAGCSRNGNRRPAPDELASLEKDFGGRIGVYALDTGSGDTVG
51398     MISRRALLVGGVSAVGVVAAGCSRNGNRRPAPDELASLEKDFGGRIGVYALDTGSGDTVG
51409     MISRRALLVGGVSAVGVVAAGCSRNGNRRPAPDELASLEKDFGGRIGVYALDTGSGDTVG
51399     MISRRALLVGGVSAVGVVAAGCSRNGNRRPAPDELASLEKDFGGRIGVYALDTGSGDTVG
51401     MISRRALLVGGVSAVGVVAAGCSRNGNRRPAPDELASLEKDFGGRIGVYALDTGSGDTVG
51415     MISRRALLVGGVSAVGVVAAGCSRNGNRRPAPDELASLEKDFGGRIGVYALDTGSGDTVG
51421     MISRRALLVGGVSAVGVVAAGCSRNGNRRPAPDELASLEKDFGGRIGVYALDTGSGDTVG
51414     MISRRALLVGGVSAVGVVAAGCSRNGNRRPAPDELASLEKDFGGRIGVYALDTGSGDTVG
51413     MISRRALLVGGVSAVGVVAAGCSRNGNRRPAPDELASLEKDFGGRIGVYALDTGSGDTVG
51418     MISRRALLVGGVSAVGVVAAGCSRNGNRRPAPDELASLEKDFGGRIGVYALDTGSGDTVG
51402     MISRRALLVGGVSAVGVVAAGCSRNGNRRPAPDELASLEKDFGGRIGVYALDTGSGDTVG
51396     MISRRALLVGGVSAVGVVAAGCSRNGNRRPAPDELASLEKDFGGRIGVYALDTGSGDTVG
51419     MISRRALLVGGVSAVGVVAAGCSRNGNRRPAPDELASLEKDFGGRIGVYALDTGSGDTVG
50937     MISRRALLVGGVSAAGVVAAGCSRNGNRRPAPDELASLEKDFGGRIGVYALDTGSGDTVG
51422     MISRRALLVGGVSAVGVVAAGCSRNGNRRPAPDELASLEKDFGGRIGVYALDTGSGDTVG
51417     MISRRALLVGGVSAVGVVAAGCSRNGNRRPAPDELASLEKDFGGRIGVYALDTGSGDTVG
51411     MISRRALLVGGVSAVGVVAAGCSRNGNRRPAPDELASLEKDFGGRIGVYALDTGSGDTVG
51410     MISRRALLVGGVSAVGVVAAGCSRNGNRRPAPDELASLEKDFGGRIGVYALDTGSGDTVG
51406     MISRRALLVGGVSAVGVVAAGCSRNGNRRPAPDELASLEKDFGGRIGVYALDTGSGDTVG
51405     MISRRALLVGGVSAVGVVAAGCSRNGNRRPAPDELASLEKDFGGRIGVYALDTGSGDTVG
51404     MISRRALLVGGVSAVGVVAAGCSRNGNRRPAPNELASLEKDFGGRIGVYALDTGSGDTVG
51425     MISRRTLLVGGVSAVGVVAAGCSRNDNRRPAPNELTSLEKDFGGRIGAYALDTGSGDTVG
47350     MISRRTLLVGGVSAGGVVAAGCSRNGNRRPAPNELTSLEKDFGGRIGVYALDTGSGDTVG
51420     MISRRTLLVGGVSAVGVVAAGCSRNGNRRPAPNELTSLEKDFGGRIGVYALDTGSGDTVG
51403     MISRRTLLVGGVSAVGVVAAGCSRNGNRRPAPNELTSLEKDFGGRIGVYALDTGSGDTVG

                   70        80        90       100       110       120
          ....|....|....|....|....|....|....|....|....|....|....|....|
ATCC19977 HRADERFLMCSTVKTFIVSAILRRRLSEPGLLDQRIQYTQSDVLEWAPITSQHVSTGMTV
51395     HRADERFLMCSTVKTFIVSAILRRRLSEPGLLDQRIQYTQSDVLEWAPITSQHVSTGMTV
51412     HRADERFLMCSTVKTFIVSAILRRRLSEPGLLDQRIQYTQSDVLEWAPITSQHVSTGMTV
51400     HRADERFLMCSTVKTFIVSAILRRRLSEPGLLDQRIQYTQSDVLEWAPITSQHVSTGMTV
51407     HRADERFLMCSTVKTFIVSAILRRRLSEPGLLDQRIQYTQSDVLEWAPITSQHVSTGMTV
50936     HRADERFLMCSTVKTFIVSAILRRRLSEPGLLDQRIQYTQSDVLEWAPITSQHVSTGMTV
51398     HRADERFLMCSTVKTFIVSAILRRRLSEPGLLDQRIQYTQSDVLEWAPITSQHVSTGMTV
51409     HRADERFLMCSTVKTFIVSAILRRRLSEPGLLDQRIQYTQSDVLEWAPITSQHVSTGMTV
51399     HRADERFLMCSTVKTFIVSAILRRRLSEPGLLDQRIQYTQSDVLEWAPITSQHVSTGMTV
51401     HRADERFLMCSTVKTFIVSAILRRRLSEPGLLDQRIQYTQSDVLEWAPITSQHVSTGMTV
51415     HRADERFLMCSTVKTFIVSAILRRRLSEPGLLDQRIQYTQSDVLEWAPITSQHVSTGMTV
51421     HRADERFLMCSTVKTFIVSAILRRRLSEPGLLDQRIQYTQSDVLEWAPITSQHVSTGMTV
51414     HRADERFLMCSTVKTFIVSAILRRRLSEPGLLDQRIQYTQSDVLEWAPITSQHVSTGMTV
51413     HRADERFLMCSTVKTFIVSAILRRRLSEPGLLDQRIQYTQSDVLEWAPITSQHVSTGMTV
51418     HRADERFLMCSTVKTFIVSAILRRRLSEPGLLDQRIQYTQSDVLEWAPITSQHVSTGMTV
51402     HRADERFLMCSTVKTFIVSAILRRRLSEPGLLDQRIQYTQSDVLEWAPITSQHVSTGMTV
51396     HRADERFLMCSTVKTFIVSAILRRRLSEPGLLDQRIQYTQSDVLEWAPITSQHVSTGMTV
51419     HRADERFLMCSTVKTFIVSAILRRRLSEPGLLDQRIQYTQSDVLEWAPITSQHVSTGMTV
50937     HRADERFLMCSTVKTFIVSAILRRRLSEPGLLDQRIQYTQSDVLEWAPITSQHVSTGMTV
51422     HRADERFLMCSTVKTFIVSAILRRRLSEPGLLDQRIQYTQSDVLEWAPITSQHVSTGMTV
51417     HRADERFLMCSTVKTFIVSAILRRRLSEPGLLDQRIQYTQSDVLEWAPITSQHVSTGMTV
51411     HRADERFLMCSTVKTFIVSAILRRRLSEPGLLDQRIQYTQSDVLEWAPITSQHVSTGMTV
```

```
51410      HRADERFLMCSTVKTFIVSAILRRRLSEPGLLDQRIQYTQSDVLEWAPITSQHVSTGMTV
51406      HRADERFLMCSTVKTFIVSAILRRRLSEPGLLDQRIQYTQSDVLEWAPITSQHVSTGMTV
51405      HRADERFLMCSTVKTFIVSAILRRRLSEPGLLDQRIQYTQSDVLEWAPITSQHVSTGMTV
51404      HRTDERFLMCSTVKTFIVSAILHRSLSEPGLLDRRIQYTQSDVLEWAPITSQHVPTGMTV
51425      HRADERFLMCSTVKTFIVSAILHRRQSEPGLLDQRIQYTQSDVLEWAPITSQHVSTGMTV
47350      HRADERFLMCSTVKTFIVSAILHRRQSEPGLLDQRIQYTQSDVLEWAPITSQHVSTGMTV
51420      HRADERFLMCSTVKTFIVSAILHRRQSEPGLLDQRIQYTQSDVLEWAPITSQHVSTGMTV
51403      HRADERFLMCSTVKTFIVSAILHRRQSEPGLLDQRIQYTQSDVLEWAPITSQHVSTGMTV

                   130       140       150       160       170       180
           ....|....|....|....|....|....|....|....|....|....|....|....|
ATCC19977  SELCDATLRYSDNTGANLLITQLGGPKETEKFVRSLGDNVTRMDRTEVQLNIPDGDLDTS
51395      SELCDATLRYSDNTGANLLITQLGGPKETEKFVRSLGDNVTRMDRTEVQLNIPDGDLDTS
51412      SELCDATLRYSDNTGANLLITQLGGPKETEKFVRSLGDNVTRMDRTEVQLNIPDGDLDTS
51400      SELCDATLRYSDNTGANLLITQLGGPKETEKFVRSLGDNVTRMDRTEVQLNIPDGDLDTS
51407      SELCDATLRYSDNTGANLLITQLGGPKETEKFVRSLGDNVTRMDRTEVQLNIPDGDLDTS
50936      SELCDATLRYSDNTGANLLITQLGGPKETEKFVRSLGDNVTRMDRTEVQLNIPDGDLDTS
51398      SELCDATLRYSDNTGANLLITQLGGPKETEKFVRSLGDNVTRMDRTEVQLNIPDGDLDTS
51409      SELCDATLRYSDNTGANLLITQLGGPKETEKFVRSLGDNVTRMDRTEVQLNIPDGDLDTS
51399      SELCDATLRYSDNTGANLLITQLGGPKETEKFVRSLGDNVTRMDRTEVQLNIPDGDLDTS
51401      SELCDATLRYSDNTGANLLITQLGGPKETEKFVRSLGDNVTRMDRTEVQLNIPDGDLDTS
51415      SELCDATLRYSDNTGANLLITQLGGPKETEKFVRSLGDNVTRMDRTEVQLNIPDGDLDTS
51421      SELCDATLRYSDNTGANLLITQLGGPKETEKFVRSLGDNVTRMDRTEVQLNIPDGDLDTS
51414      SELCDATLRYSDNTGANLLITQLGGPKETEKFVRSLGDNVTRMDRTEVQLNIPDGDLDTS
51413      SELCDATLRYSDNTGANLLITQLGGPKETEKFVRSLGDNVTRMDRTEVQLNIPDGDLDTS
51418      SELCDATLRYSDNTGANLLITQLGGPKETEKFVRSLGDNVTRMDRTEVQLNIPDGDLDTS
51402      SELCDATLRYSDNTGANLLITQLGGPKETEKFVRSLGDNVTRMDRTEVQLNIPDGDLDTS
51396      SELCDATLRYSDNTGANLLITQLGGPKETEKFVRSLGDNVTRMDRTEVQLNIPDGDLDTS
51419      SELCDATLRYSDNTGANLLITQLGGPKETEKFVRSLGDNVTRMDRTEVQLNIPDGDLDTS
50937      SELCDATLRYSDNTGANLLITQLGGPKETEKFVRSLGDNVTRMDRTEVQLNIPDGDLDTS
51422      SELCDATLRYSDNTGANLLITQLGGPKETEKFVRSLGDNVTRMDRTEVQLNIPDGDLDTS
51417      SELCDATLRYSDNTGANLLIAQLGGPKETEKFVRSLGDNVTRMDRTEVQLNIPDGDLDTS
51411      SELCDATLRYSDNTGANLLIAQLGGPKETEKFVRSLGDNVTRMDRTEVQLNIPDGDLDTS
51410      SELCDATLRYSDNTGANLLIAQLGGPKETEKFVRSLGDNVTRMDRTEVQLNIPDGDLDTS
51406      SELCDATLRYSDNTGANLLIAQLGGPKETEKFVRSLGDNVTRMDRTEVQLNIPDGDLDTS
51405      SELCDATLRYSDNTGANLLIAQLGGPKETEKFVRSLGDNVTRMDRTEVQLNIPDGDLDTS
51404      SELCDATLRYSDNTGANLLIAQLGGPKETEKFVRSLGDNVSRMDRTEVQLNIPDGDLDTS
51425      SELCDATLRYSDNTGANLLIAQLGGPKETEKFVRSLGDNVSRMDRTEVQLNIPDGDLDTS
47350      SELCDATLRYSDNTGANLLIAQLGGPKETEKFVRSLGDNVSRMDRTEVQLNIPDGDLDTS
51420      SELCDATLRYSDNTGANLLIAQLGGPKETEKFVRSLGDNVSRMDRTEVQLNIPDGDLDTS
51403      SELCDATLRYSDNTGANLLIAQLGGPKETEKFVRSLGDNVSRMDRTEVQLNIPDGDLDTS

                   190       200       210       220       230       240
           ....|....|....|....|....|....|....|....|....|....|....|....|
ATCC19977  TPQQLVANLRRLVLDEGLDSRGRDLLTDWLKRNTTGDQSIRAAVPAGWTVADKTGGGFKG
51395      TPQQLVANLRRLVLDEGLDSRGRDLLTDWLKRNTTGDQSIRAAVPAGWTVADKTGGGFKG
51412      TPQQLVANLRRLVLDEGLDSRGRDLLTDWLKRNTTGDQSIRAAVPAGWTVADKTGGGFKG
51400      TPQQLVANLRRLVLDEGLDSRGRDLLTDWLKRNTTGDQSIRAAVPAGWTVADKTGGGFKG
51407      TPQQLVANLRRLVLDEGLDSRGRDLLTDWLKRNTTGDQSIRAAVPAGWTVADKTGGGFKG
50936      TPQQLVANLRRLVLDEGLDSRGRDLLTDWLKRNTTGDQSIRAAVPAGWTVADKTGGGFKG
51398      TPQQLVANLRRLVLDEGLDSRGRDLLTDWLKRNTTGDQSIRAAVPAGWTVADKTGGGFKG
51409      TPQQLVANLRRLVLDEGLDSRGRDLLTDWLKRNTTGDQSIRAAVPAGWTVADKTGGGFKG
51399      TPQQLVANLRRLVLDEGLDSRGRDLLTDWLKRNTTGDQSIRAAVPAGWTVADKTGGGFKG
51401      TPQQLVANLRRLVLDEGLDSRGRDLLTDWLKRNTTGDQSIRAAVPAGWTVADKTGGGFKG
51415      TPQQLVANLRRLVLDEGLDSRGRDLLTDWLKRNTTGDQSIRAAVPAGWTVADKTGGGFKG
51421      TPQQLVANLRRLVLDEGLDSRGRDLLTDWLKRNTTGDQSIRAAVPAGWTVADKTGGGFKG
51414      TPQQLVANLRRLVLDEGLDSRGRDLLTDWLKRNTTGDQSIRAAVPAGWTVADKTGGGFKG
```

```
51413      TPQQLVANLRRLVLDEGLDSRGRDLLTDWLKRNTTGDQSIRAAVPAGWTVADKTGGGFKG
51418      TPQQLVANLRRLVLDEGLDSRGRDLLTDWLKRNTTGDQSIRAAVPAGWTVADKTGGGFKG
51402      TPQQLVANLRRLVLDEGLDSRGRDLLTDWLKRNTTGDQSIRAAVPAGWTVADKTGGGFKG
51396      TPQQLVANLRRLVLDEGLDSRGRDLLTDWLKRNTTGDQSIRAAVPAGWTVADKTGGGFKG
51419      TPQQLVANLRRLVLDEGLDSRGRDLLTDWLKRNTTGDQSIRAAVPAGWTVADKTGGGFKG
50937      TPQQLVANLRRLVLDEGLDSRGRDLLTDWLKRNTTGDQSIRAAVPAGWTVADKTGGGFKG
51422      TPKQLVANLRRLVLDEGLDSRGRDLLTDWLKRNTTGDQSIRAAVPAGWTVADKTGGGFKG
51417      TPQQLVANLRRLVLDEGLDSRGRDLLTDWLKRNTTGDQSIRAAVPAGWTVADKTGGGFKG
51411      TPQQLVANLRRLVLDEGLDSRGRDLLTDWLKRNTTGDQSIRAAVPAGWTVADKTGGGFKG
51410      TPQQLVANLRRLVLDEGLDSRGRDLLTDWLKRNTTGDQSIRAAVPAGWTVADKTGGGFKG
51406      TPQQLVANLRRLVLDEGLDSRGRDLLTDWLKRNTTGDQSIRAAVPAGWTVADKTGGGFKG
51405      TPQQLVANLRRLVLDEGLDSRGRDLLTDWLKRNTTGDQSIRAAVPAGWTVADKTGGGFKG
51404      TPQQLVANLRRLVLDEGLDSRGRDLLTDWLKRNTTGDQSIRAAVPAGWTVADKTGGGFKG
51425      TPQQLVANLRRLVLDEGLDSGGRDLLTDWLKRNTTGDQSIRAAVPAGWTVADKTGGGFKG
47350      TPQQLVANLRRLVLDEGLDSGGRDLLTDWLKRNTTGDQSIRAAVPAGWTVADKTGGGFKG
51420      TPQQLVANLRRLVLDEGLDSGGRDLLTDWLKRNTTGDQSIRAAVPAGWTVADKTGGGFKG
51403      TPQQLVANLRRLVLDEGLDSGGRDLLTDWLKRNTTGDQSIRAAVPAGWTVADKTGGGFKG

                   250       260       270       280
           ....|....|....|....|....|....|....|....|....|....
ATCC19977  ETNDIAVIWPPGRAPIVMAVLTVPEDPTSTKGKPTIAAATRIVLRAFGA
51395      ETNDIAVIWPPGRAPIVMAVLTVPEDPTSTKGKPTIAAATRIVLRAFGA
51412      ETNDIAVIWPPGRAPIVMAVLTVPEDPTSTKGKPTIAAATRIVLRAFGA
51400      ETNDIAVIWPPGRAPIVMAVLTVPEDPTSTKGKPTIAAATRIVLRAFGA
51407      ETNDIAVIWPPGRAPIVMAVLTVPEDPTSTKGKPTIAAATRIVLRAFGA
50936      ETNDIAVIWPPGRAPIVMAVLTVPEDPTSTKGKPTIAAATRIVLRAFGA
51398      ETNDIAVIWPPGRAPIVMAVLTVPEDPTSTKGKPTIAAATRIVLRAFGA
51409      ETNDIAVIWPPGRAPIVMAVLTVPEDPTSTKGKPTIAAATRIVLRAFGA
51399      ETNDIAVIWPPGRAPIVMAVLTVPEDPTSTKGKPTIAAATRIVLRAFGA
51401      ETNDIAVIWPPGRAPIVMAVLTVPEDPTSTKGKPTIAAATRIVLRAFGA
51415      ETNDIAVIWPPGRAPIVMAVLTVPEDPTSTKGKPTIAAATRIVLRAFGA
51421      ETNDIAVIWPPGRAPIVMAVLTVPEDPTSTKGKPTIAAATRIVLRAFGA
51414      ETNDIAVIWPPGRAPIVMAVLTVPEDPTSTKGKPTIAAATRIVLRAFGA
51413      ETNDIAVIWPPGRAPIVMAVLTVPEDPTSTKGKPTIAAATRIVLRAFGA
51418      ETNDIAVIWPPGRAPIVMAVLTVPEDPTSTKGKPTIAAATRIVLRAFGA
51402      ETNDIAVIWPPGRAPIVMAVLTVPEDPTSTKGKPTIAAATRIVLRAFGA
51396      ETNDIAVIWPPGRAPIVMAVLTVPEDPTSTKGKPTIAAATRIVLRAFGA
51419      ETNDIAVIWPPGRAPIVMAVLTVPEDPTSTKGKPTIAAATRIVLRAFGA
50937      ETNDIAVIWPPGRAPIVMAVLTVPEDPTSTKGKPTIAAATRIVLRAFGA
51422      ETNDIAVIWPPGRAPIVMAVLTVPEDPTSTKGKPTIAAATRIVLRAFGA
51417      ETNDIAVIWPPGRAPIVMAVLTVPEDLTSTKGKPTIAAATRIVLRAFGA
51411      ETNDIAVIWPPGRAPIVMAVLTVPEDPTSTKGKPTIAAATRIVLRAFGA
51410      ETNDIAVIWPPGRAPIVMAVLTVPEDPTSTKGKPTIAAATRIVLRAFGA
51406      ETNDIAVIWPPGRAPIVMAVLTVPEDPTSTKGKPTIAAATRIVLRAFGA
51405      ETNDIAVIWPPGRAPIVMAVLTVPEDPTSTKGKPTIAAATRIVLRAFGA
51404      ETNDIAVIWPPDRAPIVMAVLTVPEDPTSTKGKPTIAAAARIVLRAFGA
51425      ETNDIAVIWPPDRAPIVMAVLTVPEDPTSTKGKPTIAAAARIALRAFGA
47350      ETNDIAVIWPPDRAPIVMAVLTVPEDPTSTKGKPTIAAAARIALRAFGA
51420      ETNDIAVIWPPDRAPIVMAVLTVPEDPTSTKGKPTIAAAARIALRAFGA
51403      ETNDIAVIWPPDRAPIVMAVLTVPEDPTSTKGKPTIAAAARIALRAFGA
```

Fig S1. Alignment of Bla_{MAB} amino acid sequences from 30 *M. abscessus* complex isolates
