# Supplementary material for: Dual β-Lactam Combinations Highly Active against Mycobacterium abscessus Complex In Vitro
Source: mBio. 2019 Feb 12;10(1):e02895-18. doi: 10.1128/mBio.02895-18 (PMC6372805; doi:10.1128/mBio.02895-18)
Supplement: TABLE S1 [file mBio.02895-18-st001.docx]

Table S1. Primers used in this study

| Primer | Sequence |
| --- | --- |
| MAB16s F | CGCAACCCTTGTCCTATGTT |
| MAB16s R | CCCTCTGTACTGGCCATTGT |
| MAB2874_F | GCTTCCAAGACCATCGAGAA |
| MAB2874_R | GCTTTGGCGTTATCCAAGAC |
| blaMAB –F | GAGTTGGGTGATCAGCAGGT |
| blaMAB –R | AGTACACGCAATCCGACGTT |
